# Photothermal Catalysis of Cellulose to Prepare Levulinic Acid-Rich Bio-Oil

**DOI:** 10.3390/polym17070857

**Published:** 2025-03-23

**Authors:** Bolun Li, Mengyan Wang, Huixiang Luo, Kaina Li, Yanlong Jia, Mingjie Fu, Chenyu Jiang, Shuangquan Yao, Yongjun Yin

**Affiliations:** Guangxi Key Laboratory of Clean Pulp & Papermaking and Pollution Control, School of Light Industry and Food Engineering, Guangxi University, Nanning 530004, China; 18342278677@163.com (B.L.); 2216391044@st.gxu.edu.cn (M.W.); 2316301030@st.gxu.edu.cn (H.L.); lkn7828@163.com (K.L.); 2116391013@st.gxu.edu.cn (Y.J.); fu1354433757@163.com (M.F.); 2416301020@st.gxu.edu.cn (C.J.)

**Keywords:** photothermal catalysis, cellulose, levulinic acid, Pt/TiO_2_-CNT, bio-oil

## Abstract

As a carbon-neutral and renewable raw material, cellulose can be transformed into biomass fuels to reduce the dependence on fossil fuels and carbon dioxide emissions. In view of harsh reaction conditions, low selectivity of product, and easy deactivation of the catalyst, this study studied the use of photothermal catalytic technology to convert cellulose into bio-oil rich in levulinic acid. It was discovered that a synergistic effect between heating and photocatalysis is present in cellulose degradation. Different metals were loaded on carbon nanotubes doped with titanium dioxide to prepare different photothermal catalysts, and their catalytic effects on cellulose were compared. It was found that TiO_2_-CNT loaded with platinum metal exhibited the highest catalytic performance. By adopting Pt/TiO_2_-CNT as the catalyst, the conversion rate of bio-oil reached 99.44%, and the selectivity of LA reached 44.41% at 220 °C for 3 h. As the photothermal catalysis increased the H/C ratio and decreased the O/C ratio of the liquid product, the calorific value reached 21.01 MJ/kg. This study can promote the further industrial application of lignocellulose to prepare fuel oil and decrease the environmental pollution caused by the massive consumption of fossil fuels.

## 1. Introduction

Biomass energy, as a renewable energy source, can not only reduce dependence on fossil fuels and environmental pollution but also provide an effective carbon reduction path for industrial production fields [1]. Cellulose accounts for 30% to 50% of the lignocellulose components and is environmentally friendly and sustainable and has very extensive sources. Compared to using starch, using cellulose to produce biomass fuel has a significant cost advantage, as it does not compete with edible resources [2]. In addition, cellulose is a carbon-neutral raw material. The use of biomass fuels will reduce the demand for fossil fuels and mitigate climate change. Many researchers have studied the conversion of cellulose into liquid biofuels, including methods such as thermal cracking [3], acid or alkali depolymerization [3,4,5,6], hydrothermal liquefaction [7], etc. Cellulose can be converted into high calorific value fuel monomers such as 5-hydroxymethylfurfural (5-HMF) [3,4,5], levulinic acid (LA) [6], and γ-valerolactone (GVL) [4]. However, harsh reaction conditions, too many side reactions, few active sites, and easy deactivation of the catalyst lead to high production costs, low product selectivity, and complex processes, which limit their application in industry.

Photothermal catalysis has many advantages, such as simple operation, mild reaction conditions, low energy consumption, low cost, and high product selectivity. It presents a high level of sustainability in biomass treatment [8]. The research on photothermal catalytic conversion of cellulose into bio-oil will help reduce the cost and promote the industrial application of biomass fuel. Cellulose is a polysaccharide composed of six-carbon and five-carbon monosaccharide structural units and is the main contributor to the bio-oil from biomass pyrolysis [9]. The O/C and H/C ratios reflect the chemical reactions that occur in the conversion of biomass resources into liquid fuels [2] and determine the quality of biofuels [10]. Considering that the O/C and H/C ratios of organic acids are similar to those of biomass resources, converting cellulose into bio-oil containing organic acids is considered a very promising way to compete with the current petroleum route [1].

The dehydration reaction of cellulose can produce furfural, and furfural can be further pyrolyzed to prepare bio-oil that mainly contains γ-valerolactone [11,12], furan compounds and levoglucosone [3], sorbitol [2,13], olefins, alcohols [4], and esters [14]. Cellulose will undergo C-C bond cleavage under high temperature and acid or alkali conditions, leading to its conversion into biofuels rich in acids, aldehydes, ketones, esters, and carbohydrates. It provides a basis for the conversion of cellulose into bio-oil under photothermal conditions. Due to the dense microcrystalline structure of cellulose, its conversion path is complex, the conditions are harsh, and the selectivity is low [2]. Compared with traditional gasoline and diesel, bio-oil has a high oxygen content (45–50%), high water content, and low calorific value and thermal stability [15]. Photocatalysis can selectively cleave or functionalize the target chemical bonds and specific functional groups under mild conditions [8]. The photocatalytic process promotes the oxidation and decomposition of water to generate H^+^ and ·OH radicals. The ·OH radicals can attack cellulose and oxidize it to produce CO_2_ and H^+^. The H^+^ can then react with electrons (e^−^) generated by light to ultimately produce H_2_ [15]. Under photocatalytic conditions, cellulose undergoes C-C bond cleavage, providing a basis for its conversion into high-value-added products. However, weak light absorption, high recombination of photogenerated electrons, high pore complexation rate, and low semiconductor stability [16] lead to low conversion rate, which greatly limits its large-scale application.

To utilize the near-infrared region of the solar spectrum, researchers have proposed photothermal catalysis, which has been confirmed as an effective photocatalytic strategy [17]. Wang selectively converted cellulose into bio-oil rich in 5-HMF (accounting for 48.4%) at relatively low temperature (120 °C) and light (300 W Xe lamp). Their research also points out that converting cellulose into various valuable products by photothermal catalysis involves complex mechanisms, including oxidation, C-C bond cleavage, and photochemical reactions. Developing efficient photothermal catalytic systems to selectively cleave C-O and C-C bonds in natural cellulose is an important research direction in this field [1].

LA is the “core bridge” between biomass raw materials and high-value hydrocarbon fuels, which can be converted into bio-liquid fuel. Its content and properties reflect the quality of bio-oil and the advantages and disadvantages of the production process [18]. Considering that there are some issues in producing LA from cellulose, such as difficult depolymerization of cellulose structure, low yield of target products, and easy blockage and deactivation of the catalyst, it is very important to develop efficient catalytic reaction systems for improving the yield of target products and the quality of bio-oil fuels. Molecular sieves and solid acid are mainly catalysts for promoting the conversion of cellulose to bio-oil. As a typical Lewis acid catalyst, ZnCl_2_ promotes the depolymerization of cellulose and hemicellulose and generates more furfural, but the catalyst is corrosive and difficult to recycle [19]. Pan et al. used CoCl_2_ as a catalyst to construct a homogeneous Lewis acid catalytic system, converting cellulose into bio-oil with a maximum yield of 22.8% [20]. Titanium dioxide (TiO_2_) is the most widely used photocatalyst in the catalytic oxidative depolymerization of cellulose due to its high activity, chemical stability, and low cost. Zhang used TiO_2_ to convert cellulose into sugar and carbon dioxide under ultraviolet or sunlight irradiation and provided the possibility of recycling sugar as a liquid fuel [21]. However, the band gap of TiO_2_ is 3.2 eV, which can only absorb the ultraviolet with a wavelength less than 380 nm. Therefore, the photocatalytic efficiency of TiO_2_ under sunlight is low, which limits its practical application. Metal oxides are widely used as solid acid catalysts to promote the hydrolysis of cellulose and the thermal catalytic conversion of cellulose into glucose. Heng reported the photocatalytic degradation of cellulose using TiO_2_-Au, selectively producing arabinose and gaseous fuels through a direct C_1_-C_2_α-cleavage mechanism [22]. Meanwhile, a carbon nanotube (CNT) can form multi-channel electron transport channels, which can promote the transfer of photogenerated electrons and reduce the opportunity for electron hole recombination. CNTs are the ideal catalyst support because of their unique hollow structure, high specific surface area, and strong adsorption capacity [23]. CNTs also have a certain preparation technology, which gives the catalyst advantages in resource utilization. TiO_2_ is rich in resources and does not require high temperature and high pressure for preparation. TiO_2_ has been identified as the most promising photocatalytic material based on its chemical stability [24]. Therefore, it is very promising to use metal oxides with thermal effects and CNTs to achieve solar-driven depolymerization of cellulose.

To change the harsh operating conditions, reduce the produce cost, and improve the conversion rate and product selectivity of bio-oil, photothermal catalytic technology for producing bio-oil rich in LA from cellulose was studied in this study. Different metals were loaded on TiO_2_-doped CNTs (TiO_2_-CNT) to prepare different photothermal catalysts, and the conversion rate and LA selectivity of products were used as indicators to screen the photocatalyst. The photothermal catalytic process condition and the reaction mechanism were discussed. This study will promote the further industrial application of lignocellulose to produce fuel oil.

## 2. Materials and Methods

### 2.1. Materials and Reagents

Cellulose ((C_6_H_10_O_5_)n, AR, ≤25 μm), copper sulfate (CuSO_4_, AR, 99%), chloroplatinic acid hydrate (H_2_Cl_6_Pt · xH_2_O, AR, 99.95%), ammonium metavanadate (NH_4_VO_3_, AR, 99%), palladium chloride (PdCl_2_, AR, 98%), silver nitrate (AgNO_3_, AR, 99%), gold chloride (AuCl_3_, AR, 99.9%), ruthenium acetate (C_14_H_21_O_15_Ru_3_, 110 AR, 48%), titanium dioxide nanoparticles (TiO_2_, anatase, AR, 99.8%, <5 nm), and multi-walled carbon nanotubes (CNT, AR, >95%, outer diameter: 8–15nm, length:~50 μm, SSA: >140m^2^/g) were purchased from Aladdin Pharmaceuticals Inc. (Riverside, CA, USA), H_2_SO_4_ (AR, 95%), NaOH (AR, 97%), Na_2_SO_4_ (AR, >99%), ethyl acetate (AR, >99%), ethanol (C_2_H_5_OH, AR, >99.7%), and cyclohexane (C_6_H_12_, AR, >99.5%) were purchased from Nanning Boyu Laboratory Equipment Co., Ltd. (Nanning, China).

### 2.2. Experimental Method

#### 2.2.1. Preparation of Catalyst

The catalyst was prepared by a two-step synthesis method of alkaline hydrothermal and metal ion exchange [25]. Firstly, 5 g nano-TiO_2_ were dispersed into 150 mL 10 M NaOH aqueous solution and stirred for 24 h, and then the sample was transferred to an autoclave lined with Teflon. After heating at 150 °C for 48 h, the sample was washed with 0.1 mol dilute nitric acid solution and deionized water to a neutral state. The sample was placed in a vacuum drying oven at 60 °C for 24 h, then taken out and ground. A small amount of CNT was placed in 25 mL concentrated nitric acid and refluxed at 80 °C for 6 h. After the reactants were cooled naturally and washed and filtered 6 times, they were dried at 80 °C for 24 h. Then, the treated nano-TiO_2_ was mixed with the acid-washed CNT, and anhydrous ethanol and deionized water were added, stirred for 1 h, and ultrasonically shocked for 1 h to obtain a uniformly dispersed black solution. After filtration, the solution was placed in a vacuum drying oven at 60 °C for 24 h, then taken out and ground. After mixing TiO_2_-CNT with a certain amount of metal compound M for 24 h, the samples were dried in an oven at 80 °C for 12 h and calcined at 400 °C for 3 h in a muffle furnace. Then, they were heated to 450 °C at a heating rate of 5 °C/min in a tube furnace and reduced with a 10% H_2_/Ar_2_ mixed gas flow at 25 mL/min for 6 h. The catalyst M/TiO_2_-CNT was obtained after the above treatment.

#### 2.2.2. Photothermal Catalysis Experiment

The photothermal catalytic experiment of cellulose was carried out by a photothermal reactor with photocatalytic xenon lamp (CEL-HKT250, Zhongjiao Jinyuan Technology Co., Ltd., Beijing, China). Cellulose, the photothermal catalyst, and 5% acetic acid (the solvent system) were loaded into a 100 mL quartz-lined reactor, and N_2_ was used to empty the air before the reaction. The reaction temperature, stirring speed, and reaction time were set to start the reaction. As the reaction was completed, the products in the reactor were filtered to separate the solid and liquid, the organic solvent was removed by vacuum distillation, and the bio-oil was obtained.

### 2.3. Catalyst Characterization and Product Detection

#### 2.3.1. Characterization and Analysis of the Catalyst

The specific surface area and pore size distribution of the catalyst were analyzed by automatic specific surface and porosity analyzer BET (ASAP 2460, Micromeritics, Atlanta, GA, USA). The valence state of the elements in the catalyst was analyzed by X-ray photoelectron spectroscopy (ESCALAB 250 XI+, Thermo Fisher Scientific, Waltham, MA, USA), and the C1’s peak at 284.8 eV was collected to determine the binding energy (BE) value. The crystal structure of the catalyst was analyzed by X-ray diffractometer (Rigaku D/MAX 2500V, Nippon Science Corporation, Tokyo, Japan). The apparent morphology of the catalyst was analyzed by a 300 kV field emission transmission electron microscope (TECNAI G2 F30, FEI, Hillsborough, OR, USA). The UV-visible absorption spectra of the catalyst were analyzed by UV spectrophotometer (LAMBDA 950, PE, Hayward, CA, USA). The characteristic absorption intensity of the catalyst in the visible light range was observed by scanning the full spectrum of the catalyst at 300–900 nm. The stability of the catalyst was analyzed by a thermogravimetric analyzer (STA 449F5, NETZSCH, Seerbu, Germany).

#### 2.3.2. Detection and Analysis of Bio-Oil

The element contents of C, H, O, N, and S in the cellulose and bio-oil were measured by an element analyzer (Vario EL cube, Elementar Analysensystem, DE, Frankfurt, Germany). The high heat value (HHV) of the liquid fuel was calculated by the Dulong formula [26], as follows:

(1) $$HHV\left(\frac{MJ}{kg}\right)=0.335\times M_{c}+1.42\times M_{H}-0.154\times M_{O}-0.145\times M_{N}$$

Here, M_C_, M_H_, M_O_, and M_N_ represent the mass percentages (%) of C, H, O, and N in organic matter, respectively.

The liquid products were analyzed by a Fourier transform infrared spectrometer (IRTracer-100, Shimadzu, Tokyo, Japan) with a spectral range of 4000–400 cm^−1^ and a resolution of 4 cm^−1^. The liquid product components were measured by gas chromatography–mass spectrometry (7890A-5975C, Agilent Technologies, Santa Clara, CA, USA), and the peak area normalization scanning method was used for qualitative and quantitative analysis of bio-oil [27], Equipped with a capillary chromatographic column (HP-5MS, 30 m × 0.25 mm × 0.25 μm). The GC heating program was as follows: initially, the column temperature was maintained at 80 °C, then increased to 180 °C at a rate of 5 °C/min (maintaining for 10 min) and finally increased to 280 °C at a rate of 5 °C/min (maintaining for 5 min). The components of the bio-oil were tested by a Nuclear Magnetic Resonance (NMR) Spectrometer (Avance III HD500 MHz, Bruker, Karlsruhe, Germany). The HSQC nuclear magnetic resonance spectrum of ^1^H-^13^C was obtained using a phase-sensitive gradient edited HSQC program (Ghsqcad). The data processing was carried out using Topspin software 2.1. The remaining CDCl_3_ solvent peak was used as an internal reference (δ_C_ = 77.1 ppm; δ_H_ = 7.26 ppm).

The yields of liquid products and solid products refer to the ratio of the mass of bio-oil and solid residue after catalytic degradation to the initial amount of cellulose, which can be calculated by Equations (2) and (3) [28], respectively. Considering that gasification products are very rare, their impact on products yields can be ignored [29].

(2) $$\mathrm{Yield}\;\mathrm{of}\;\mathrm{liquid}\;\mathrm{products}\;\left(\%\right)=\frac{\mathrm{the}\;\mathrm{weight}\;\mathrm{of}\;\mathrm{liquid}\;\mathrm{products}}{\mathrm{the}\;\mathrm{weight}\;\mathrm{of}\;\mathrm{initial}\;\mathrm{cellulose}}\;\times \;100\%.$$

(3) $$\mathrm{Yield}\;\mathrm{of}\;\mathrm{solid}\;\mathrm{products}\;(\%)=\frac{\mathrm{the}\;\mathrm{weight}\;\mathrm{of}\;\mathrm{solid}\;\mathrm{residues}}{\mathrm{the}\;\mathrm{weight}\;\mathrm{of}\;\mathrm{initial}\;\mathrm{cellulose}}\;\times \;100\%.$$

The mass ratio of LA to bio-oil is used to evaluate the selectivity of bio-oil products. Based on the qualitative analysis software on the GC-MS instrument (Version 10.0), the selectivity was calculated by the compound peak area normalization method. According to the ratio of the relative peak area of LA (S_X_) measured by GC-MS to the total peak area of bio-oil (ΣS_i)_, the selectivity can be calculated by Equation (4). Meanwhile, the standard curve of the levulinic acid standard solution is tested for product comparison analysis.

(4) $$W_{x}=(S_{x}∕\sum S_{i})\times \;100\%$$

## 3. Results and Discussion

**Figure 1:**
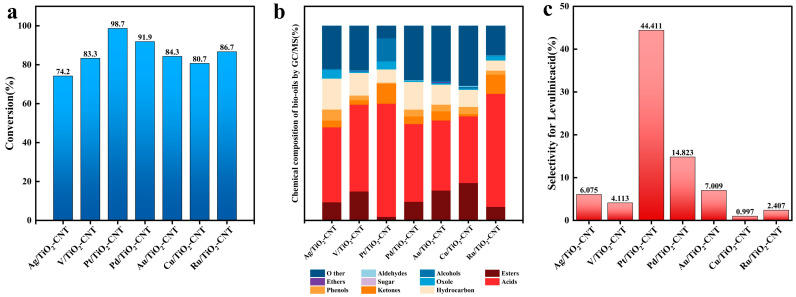
Effects of TiO_2_-CNT doped with different metal catalysts on cellulose conversion: (**a**) conversion rate; (**b**) product distribution of bio-oil; (**c**) selectivity for LA.

To clarify the superiority of photothermal catalysis in cellulose depolymerization, the differences in the degradation performance of cellulose under the same catalytic process conditions (xenon lamp light 500 W, 220 °C, 200 rpm, time 4 h, acetic acid 5%) with or without the conditions of light, heat and catalysts were investigated (a catalyst loaded with a certain metal is randomly selected). The results are shown in Table 1. To improve the degradation rate of cellulose, the effects of catalysts prepared by TiO_2_-CNT doped with different metals on cellulose degradation were analyzed to obtain the most suitable catalysts, as shown in Figure 1.

### 3.1. The Effects of Photothermal Catalysis and Catalysts on the Depolymerization of Cellulose

As shown in Table 1, cellulose does not degrade with or without light under the condition of no catalyst and no heating, but the degradation rate of cellulose can reach 70.3% by heating to 220 °C under light, which indicates that heating is necessary to maintain the high degradation rate of cellulose. As the TiO_2_-CNT catalyst was added, cellulose was partially degraded (13.6%) even without heating. The degradation rate increased 6% under the same heating conditions and increased from 70.3% to 78.1% under the same heating and light conditions, indicating that the catalyst can promote the further degradation of cellulose, and a synergistic effect between heating and photocatalysis is present in cellulose degradation. Because the hydroxyl group on the surface of CNT forms a heterojunction with titanium dioxide, which is conducive to the separation of photogenerated electron holes, it improves the quantum efficiency of titanium dioxide and increases the light absorption and carrier lifetime in the visible spectral range [30]. Therefore, the heating effect can significantly improve the photocatalytic performance, while photocatalysis (samples 5 and 7) has a limited effect on the degradation of cellulose (only 13.6% and 22.5%). Under the action of TiO_2_-CNT, the degradation rate of cellulose under photothermal catalysis is consistent with other high-temperature pyrolysis studies (77%) [2]; more than 20% of cellulose could not be utilized. As TiO_2_-CNT loaded Pt was selected as the catalyst, the photocatalytic degradation rate of cellulose increased from 13.6% to 22.5%, and the pyrolysis degradation rate (87%) and photothermal catalytic degradation rate (98%) of cellulose were significantly improved. The conversion rate of cellulose was much higher than the highest conversion rate of other studies; the conversion rate varied from 39% to 92% at higher temperatures (230 °C to 320 °C) [31]. In other studies, cellulose was completely degraded when the pyrolysis temperature reached 500 °C [32]. This is because noble metals can accelerate the separation of electron holes, effectively accumulate photogenerated electrons, and provide more reaction sites to enhance the activity of photocatalysts [33].

As shown in (Figure 1a), except for Ag/TiO_2_-CNT-loaded metals, other metals can improve the photocatalytic performance to varying degrees. The effect of these catalysts on the conversion rate of cellulose is as follows: Pt > Pd > Ru > Au > V > Cu, which is significantly higher than that of other studies (25~77%) [2]. In particular, after doping with the noble metals Pt, Ru, or Pd, the conversion rate is significantly higher than that of transition metals. Noble metals can effectively accumulate photogenerated electrons, accelerate the separation of electron holes, and provide more reaction sites to enhance the activity of the photocatalyst [33]. In addition, an appropriate amount of metal loading helps inhibit the growth of the anatase phase and increase the specific surface area of the catalyst. Noble metal deposition provides active sites, which are expected to further enhance the photocatalytic activity, thereby improving the photothermal catalytic performance [34]. Considering that the ionic radius of Ag (115 pm) is larger than that of Ti (86 pm) and there is no good loading to TiO_2_-CNT, which weakens the catalytic activity of the Ag/TiO_2_-CNT photocatalyst [35], the degradation rate of cellulose decreased from 78.1% to 74.23%. Due to the highest Schottky barrier, Pt can capture more electrons to reduce the electron hole recombination rate and obtain the highest photoreaction efficiency [33], adopting Pt/TiO_2_-CNT as the catalyst achieving the highest degradation rate of cellulose.

The volatile components of liquid fuels produced by different catalysts were analyzed by GCMS, as shown in (Figure 1b). Ten compounds can be tested, including sugars, furans, acids, ketones, esters, ethers, alcohols, aromatic compounds, hydrocarbons, and phenols. This is consistent with the results of bio-oil components measured by GCMS in the literature [36]. All the relative content of acid in cellulose pyrolysis products reaches over 30% as different catalysts are adopted. C_7_ − C_22_ accounts for 95% of hydrocarbons, which are the main components of diesel. The liquid fuel produced by Pt/TiO_2_-CNT catalyzed cellulose has the least impurities, with acids accounting for 58%, and their main component is LA, with a content as high as 44.41% (Figure 1c), followed by phenols (10.18%) and alcohols (11.87%). Pt/TiO_2_-CNT can accurately cleave the β-1,4-glycosidic bond of cellulose into glucose and then selectively cleave the C_3_ − C_4_ bond of glucose to obtain LA. This may be because Pt has more active sites than other metals, because the d-orbit electrons of platinum are in an unfilled state. At the same time, d-orbit electrons have the phenomenon of energy level interleaving, so Pt has better activity, stability, and selectivity. The liquid fuel obtained by Ag/TiO_2_-CNT catalysis contains the most hydrocarbons (C_10_ − C_29_ accounting for about 15%), and acids account for 30% (Figure 1c), of which hexadecanoic acid accounts for 24% and LA accounts for 6.08% (Figure 1c). The content of hexadecanoic acid in the liquid fuel of V/TiO_2_-CNT catalysis was 28%, and the content of LA was 4.11% (Figure 1c). The content of LA and hexadecanoic acid in the Pd/TiO_2_-CNT group was about 15%. Liquid fuel with 2.4% LA acid and 40% hexadecanoic acid can be obtained by Ru/TiO_2_-CNT catalysis, and the percentages of LA in the liquid fuel obtained by Au/TiO_2_-CNT and Cu/TiO_2_-CNT catalysis were 7.01%, and 1.00%, respectively. There is also a small number of alkanes, furans, and ketones, for example, heptadecane, octadecane, 2,5-hexanedione, etc. The long-chain organic compounds in liquid fuels are the precursors of high-density fuels, which can be further refined to prepare high-performance fuels.

It is obviously superior in conversion (42–91%) and selectivity [37,38]. Although studies have used Ru/C as a catalyst to convert 99% of cellulose into bio-oil, it takes more than 6 h [37]. This will reduce production efficiency, that is, increase production costs. A group of researchers used ZrP to catalyze cellulose at 220 °C for 4 h, and the maximum yield of LA was 28% [38]. Compared with other literature, the MCC conversion was 91.4%, and the yield of LA was 15% at 215 °C and 5 h [39]. The researchers used Ru/C to catalyze cellulose at 220 °C for 6 h, wherein catalyzation can reach 99%, but our photothermal catalysis experiment was shortened by half the time, and the same effect can still be achieved.

Considering that the best degradation rate (98.76%) and selectivity (44.41%) can be obtained as Pt/TiO_2_-CNT is adopted, Pt/TiO_2_-CNT was used as a catalyst for subsequent research.

### 3.2. Characterization and Analysis of Catalysts

**Figure 2:**
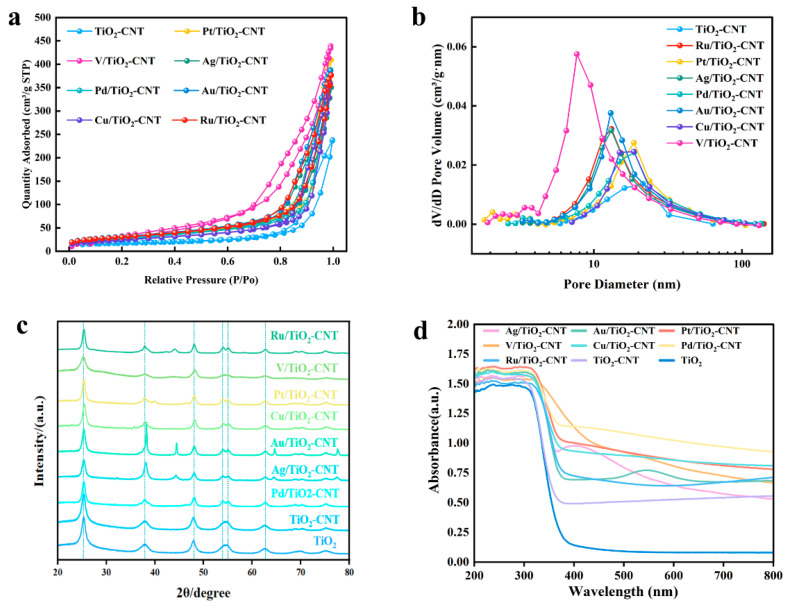
BET, XRD, and UV-vis of different catalysts: (**a**) N_2_ adsorption–desorption isotherms for catalytic materials; (**b**) pore size distribution analysis; (**c**) XRD spectra of TiO_2_-CNTphotocatalysts doped with different metals; (**d**) UV-Vis profiles of modified photocatalyst.

As shown in Figure 2a,b, all the N_2_ adsorption/desorption isotherms corresponded to type IV with H1-type hysteresis loops, indicating that all the catalysts present a mesoporous structure [40]. The embedded image of the pore size distribution indicates that the catalysts loaded with different metals have similar porous structures. The specific surface areas, pore volumes, and pore sizes of different catalysts are shown in Table 2. The high specific surface area of V/TiO_2_-CNT may be due to the increase in the number of uniformly arranged particles and catalyst particles [41]. The surface area and pore volume of TiO_2_-CNT were 60.98 m^2^/g and 0.37 cm^3^/g, respectively. According to the IUPAC classification, all the catalysts showed type II isotherms, indicating that the catalyst has a complete structure but a small surface area. Compared with TiO_2_-CNT, the catalyst loaded with Pt, Pd, and Cu metals had the large specific surface area, but the average pore size hardly changed. Large pore size and surface area can increase the contact area between reactants and catalysts and improve the reaction rate. As Ag, V, Au, or Ru metals are loaded, the pore size of the catalyst decreases. The reason may be that the ion radius of Ag (0.115 nm) and Au (0.135 nm), V (0.128 nm), and Ru (0.062 nm) is greater than the TiO_2_ radius (0.0605 nm). In addition, TiO_2_ is a porous structure, and the pores may be blocked by the loaded metal. As shown in Figure 2c, the XRD patterns of TiO_2_ doped with different metals are basically the same, and the characteristic diffraction peaks of TiO_2_ appear at 2θ = 25.40°, 38.6°, 48.04°, 53.90°, and 55.06°, corresponding to the anatase crystal phase (JCPDS card number 21-1272) [40]. No characteristic peak of CNT was found. This may be due to the overlap of the strong peaks of CNT and TiO_2_ [23]. The same anatase crystal phase can be observed by comparing the diffraction patterns of TiO_2_ doped with different metals with those of pure TiO_2_. The individual characteristic diffraction peaks of TiO_2_ doped with different metals are slightly sharp, but the TiO_2_ skeleton structure is not destroyed, indicating that the modified catalyst still maintains a relatively complete skeleton structure and does not change the original crystal structure of TiO_2_. After doping Pt, V, Ru, and Cu, the crystal plane characteristic diffraction peaks did not appear because the highly dispersed metal species on TiO_2_ lead to an unclear characteristic diffraction peak [42]. After doping Au and Ag, the characteristic diffraction peaks of the crystal plane appeared. The characteristic diffraction peaks of the Ag crystal plane appear at 44.39°, 64.58°, and 77.54°, and the diffraction peaks related to metal Au appear at 2θ = 44.4°. The reason may be that the ionic radius of Ag (0.115 nm) and the Au ion radius (0.135 nm) are larger than the radius of TiO_2_ (0.0605 nm). They have difficulty entering the lattice of TiO_2_, and a small amount of metal ions are dispersed on the surface of TiO_2_ [43]. As shown in Figure 2d, both TiO_2_-CNT and TiO_2_-CNT doped metals have an obvious red shift compared with that of TiO_2_, and the absorption in the visible light band of 400~800 nm is gradually strengthened and grows closer to visible light. Because noble metals are excellent electron capture agents, TiO_2_ is excited by light, and the generated electrons migrate to the metal Fermi level, which helps the catalyst effectively capture photogenerated electrons. It is apparent that the absorption of the catalyst within this range is caused by metal doping. Meanwhile, the energy gap of the catalyst is significantly reduced as metal is loaded, which improves the absorption capacity of the catalyst to visible light [44] and enhances its catalytic activity.

**Figure 3:**
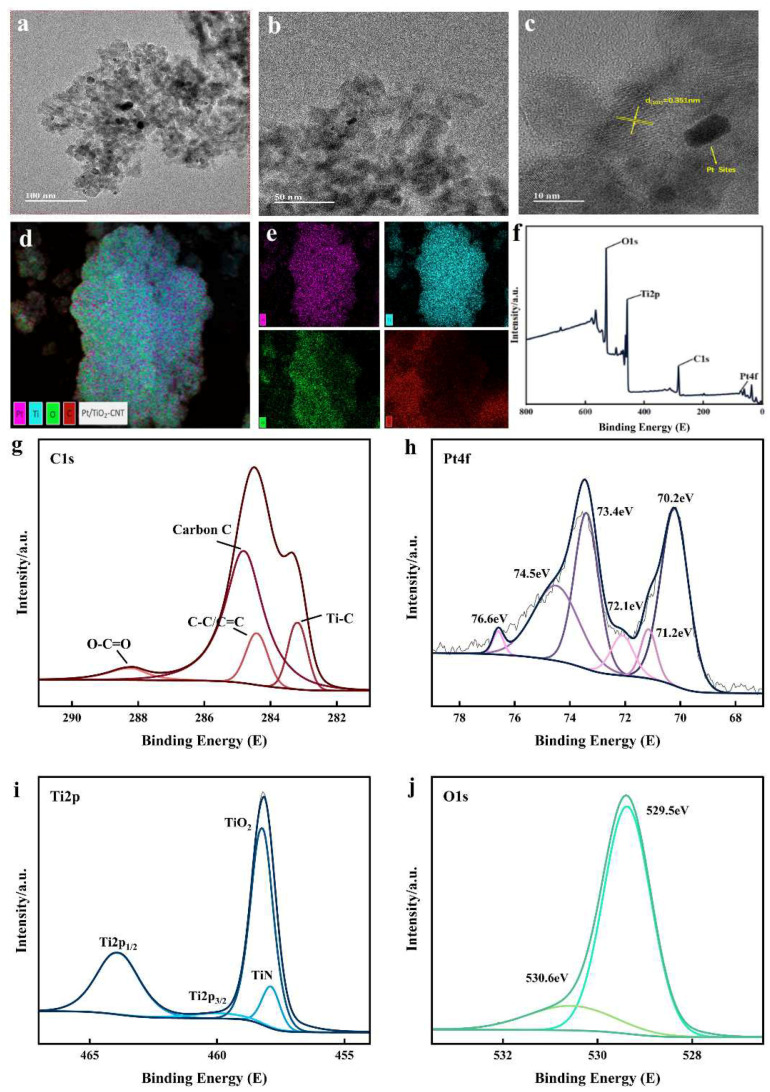
Transmission electron microscopy (TEM) images of Pt/TiO_2_-CNT catalyst: (**a**–**c**) TEM images of different sizes; (**d**,**e**) single atom and combined energy dispersive X-ray spectroscopy (EDS) images; (**f**–**j**) XPS test results for Pt/TiO_2_-CNT (**f**) full spectrum scan; (**g**) C1s; (**h**) Pt4f; (**i**) Ti2p; (**j**) O1s.

The choice of catalyst is mainly based on its degradation rate of cellulose and its selectivity to LA. Pt/TiO_2_-CNT has the best conversion effect based on high conversion rate and LA selectivity, so only the TEM of Pt/TiO_2_-CNT had been tested to confirm the successful load of metal ions. As shown in Figure 3a–c, Pt is irregularly dispersed on the surface of the catalyst, which indicates that Pt has not experienced sintering during the calcination process. The tested lattice spacing of titanium dioxide is 0.351 nm (Figure 3c), which is consistent with the lattice spacing of anatase titanium dioxide. As shown in Figure 3d,e, EDS results show that highly dispersed Pt, C, O, and Ti are observed, which indicates that there is no obvious agglomeration on the surface of TiO_2_ after doping Pt, and the dispersion is obviously improved. As a carrier, TiO_2_-CNT can improve the dispersion of Pt and improve the catalytic activity of the catalyst. Metals with small particle size and high dispersion can increase their contacts with the reactants, thereby improving the catalytic activity [45], and the dispersion of the active metal components in catalysts will affect the intermediate product and the generation of the final product [46]. Consistent with other research, the catalyst-doped Pt in the TEM image is similar to that of TiO_2_, which is consistent with the conclusion of XRD characterization that the catalyst still maintains a relatively complete skeleton structure and does not change the original crystal structure of TiO_2_ [30]. The XPS full spectrum of Pt/TiO_2_-CNT is shown in Figure 3f; the photoelectron spectroscopy peaks near 74 eV, 285 eV, 460 eV, and 530 eV correspond to the electron binding energy of Pt4f, C1s, Ti2p, and O1s, respectively. This shows that there are O, Ti, C, and Pt elements on the surface of the catalyst. Figure 3g shows the spectra collected from the C1s core level, and the characteristic peaks at the binding energies of 284.8 eV, 285 eV, and 288.2 eV correspond to aliphatic/aromatic carbon groups, amorphous carbon, carboxylic acid groups, esters, or lactones (-COOR) [47,48,49]. A peak representing the Ti-C bond appears near the binding energy of 283.0 eV and is caused by the C atom replacing the O in the TiO_2_ lattice and entering the lattice [50]. Figure 3h shows that the peaks with binding energies of 71.2 eV and 70.2 eV correspond to the metal platinum in the Pt/TiO_2_-CNT catalyst [34]. The peak with a binding energy of 73.4 eV represents Pt^2+^, which is due to the partial oxidation of the surface. The peaks with binding energies of 74.5 eV and 76.6 eV correspond to Pt^4+^ [47]. Pt exists in the form of Pt0 and Pt^4+^, which may be due to the reduction of Pt to Pt0 species and Pt^4+^ during the preparation process. This proves that there is an interaction between Pt and TiO_2_, resulting in the transfer of electrons from TiO_2_ to Pt [51]. Two broad peaks in Figure 3i reveal a high-resolution Ti 2p spectrum, in which the binding energy peaks centered at 464.7 eV and 459 eV correspond to the characteristic peaks of Ti 2p_1/2_ and Ti 2p_3/2_, indicating that the signal of titanium dioxide is not shielded by the carbon layer [52]. The peak at 458.5 eV can be attributed to the presence of TiO_2_ [34]. Figure 3j shows that the weak convolution peak with a binding energy of 530.6 eV represents the O-H bond, indicating the oxygen in the hydroxyl functional group on the surface of TiO_2_, which is beneficial for the catalyst to adsorb the degraded substrate and capture the photogenerated holes generated by photoexcitation to form hydroxyl radicals [50]. The strong convolution peak with a binding energy of 529.5 eV represents the Ti-O bond in TiO_2_, indicating the chemical state of O in the TiO_2_ lattice [53].

**Figure 4:**
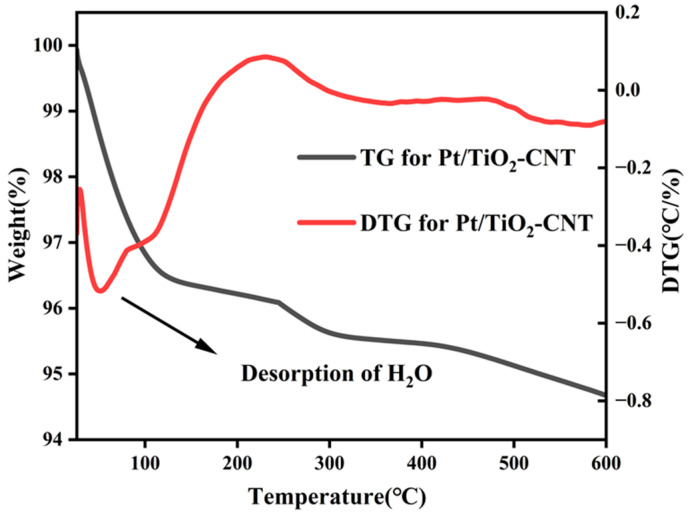
The TG-DTG curves of Pt/TiO_2_-CNT samples.

Meanwhile, considering that Pt/TiO_2_-CNT has the best conversion effect based on high conversion rate and LA selectivity, only the TG-DTG curves of Pt/TiO_2_-CNT were tested to analyze its stability as a catalyst. As shown in Figure 4, Pt/TiO_2_-CNT is stable in the test range of 100–600 °C, including the highest treatment temperature (450 °C) reached during the catalyst preparation process and the photothermal reaction temperature (220 °C). A weight loss peak occurs at 100 °C, corresponding to the loss of absorbed water, and the weight loss in the range of 100–600 °C corresponds to the absorbed carbon dioxide. This phenomenon may be due to the sample being exposed to air, absorbing some water and carbon dioxide. The weight loss of the Pt/TiO_2_-CNT catalyst was only 3.5% at 100 °C and 5.3% at 600 °C, which showed a more stable TG curve than that of Pt/TiO_2_-CNT in other studies (the weight loss was 7.1% at 300 °C) [54]. Pt/TiO_2_-CNT has a lower specific surface area than that of Pt/TiO_2_ and absorbs a small amount of CO_2_ [55].

### 3.3. Analysis of Pyrolysis Products

**Figure 5:**
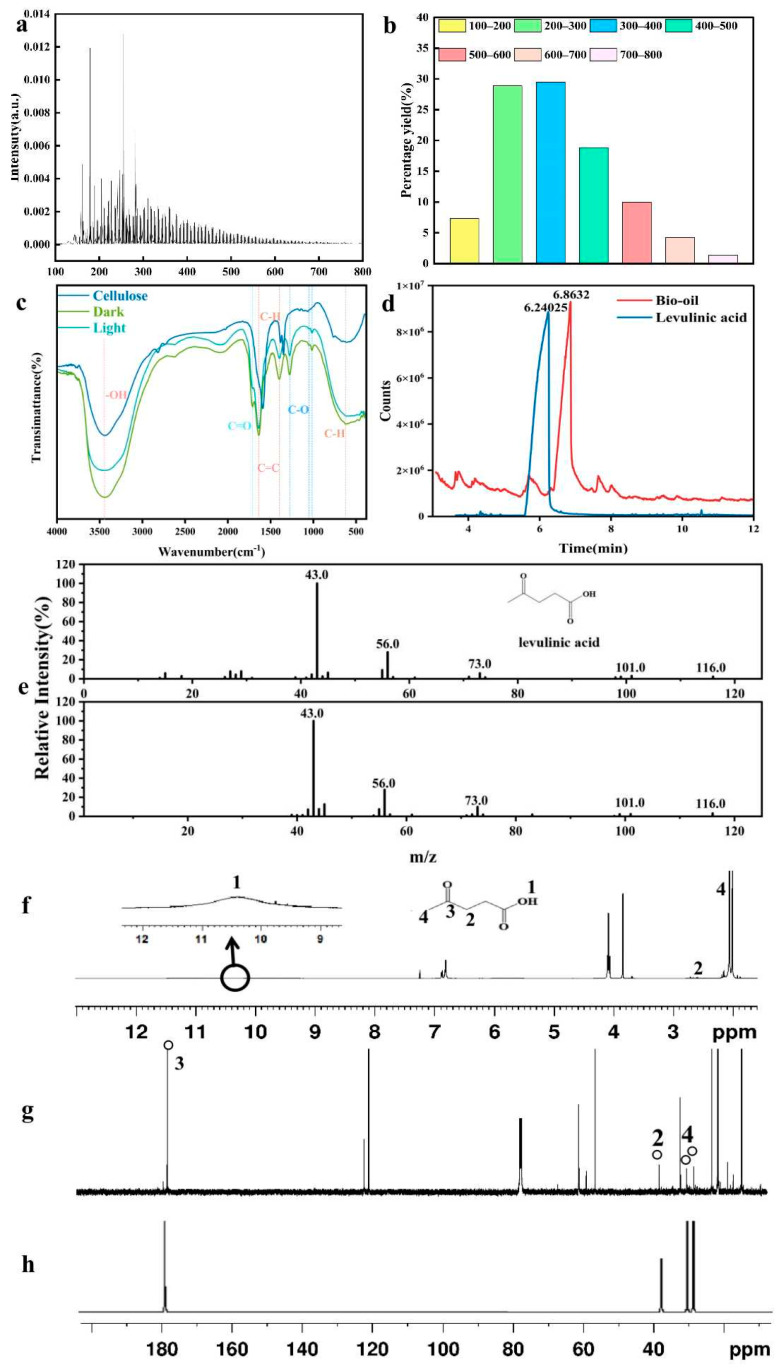
Functional groups, molecular weight, and GC-MS spectra of cellulose bio-oil: (**a**) FT-ICR-MS mass spectrum; (**b**) histogram of molecular weight distribution; (**c**) FTIR spectrum of cellulose and bio-oil (with a xenon light source and reaction in the dark); (**d**) color spectrum of bio-oil and standard levulinic acid; (**e**) mass spectra of levulinic acid standard and bio-oil; (**f**) ^1^H NMR spectra of bio-oil; (**g**) ^13^C NMR spectra of bio-oil; (**h**) standard LA.

The heavy components and volatile components of the liquid fuel are analyzed by FT-ICR-MS. As shown in Figure 5a, the molecular weight of degradation products is mainly concentrated in the range of 150–600 Da, with peaks appearing in the range of 250–300 Da. The absorption peaks of some molecules are obviously higher. These absorption peaks correspond to the substances with about 20 carbon atoms. There are very few substances with molecular weight greater than 500 Da. This is similar to the data of cellulose bio-oil measured by other researchers using FT-ICR-MS [56]. According to the molecular weight distribution analysis shown in Figure 5b, the components with molecular weights between 200 Da and 300 Da account for 28.88%, while the components with molecular weights between 300 Da and 400 Da account for 29.49%. The components with molecular weights of 400–500 Da, 500–600 Da, 600–700 Da, and 700–800 Da account for 18.84%, 9.95%, 4.25%, and 1.39%, respectively. This indicates that the content of dimers and tetramers in liquid fuels accounts for about 58%, and most of the cellulose is degraded into oligomers by photothermal catalytic depolymerization. FTIR analysis of bio-oil functional groups produced by cellulose under light (with a xenon light source) and non-light conditions (reaction in the dark) is shown in Figure 5c. Meanwhile, FTIR analysis of the raw cellulose was conducted as well, and the results are also presented in Figure 5c. The degradation products have a strong absorption peak near 3435.7 cm^−1^, mainly generated by the stretching vibration of the functional group -OH, indicating that there is water or hydroxyl compounds in bio-oil. Compared with that of the original cellulose, the stretching vibration peak of bio-oil -OH is weakened, and the hydrogen bonding of bio-oil after photothermal catalysis is reduced, indicating that hydroxyl radicals break the hydrogen bonds between and within cellulose molecules, causing the depolymerization of cellulose macromolecules [4]. There is a strong absorption peak near 1713.2 cm^−1^, mainly caused by the C=O bond stretching vibration, indicating that it contains ketones, organic acids, aldehydes, esters, and other substances [57]. According to GCMS component analysis, it may also include 2,5-hexanedione, levulinic acid, or methyl pentanoate. The absorption peak of 1638.9 cm^−1^ indicates the C=C bond stretching vibration, and the absorption peak of 622.6 cm^−1^ indicates that the C-H bond has undergone plane bending, indicating that there are aromatic compounds in the products [58]. The absorption peaks near 1276.6 cm^−1^ and 1051.8 cm^−1^, mainly caused by the C-O bond stretching vibration, indicate that there are alcohols, ethers, phenols, and other substances [59]. The characteristic absorption peaks of the pyran ring stretching vibration at 1058 and 1165 cm−1 and the C-O-C stretching vibration connected by the β -1,4-glycosidic bond of cellulose have basically disappeared, indicating that the chemical structure of cellulose undergoes drastic changes in the pyrolysis zone, β-1, and the four glycosidic bonds are almost completely broken [60]. Bio-oil produced by the photothermal catalytic of cellulose mainly contains organic acids, ketones, alcohols, aldehydes, esters, phenols, fats, and aromatics, based on inference from the infrared spectrum analysis.

As shown in Figure 5d, the peak time of levulinic acid in the standard sample is 6.24025 min, and the peak time of levulinic acid in bio-oil is 6.8632 min, because bio-oil is a complex mixture that affects the response time of levulinic acid during GC/MS analysis. This value can be accepted within 10% of the relative standard deviation [61]. As shown in Figure 5e, the mass spectra of bio-oil conform to the characteristic peaks of levulinic acid (*m*/*z* 43.0, 56.0, 73.0, 101.0, and 106.0), confirming the presence of levulinic acid in bio-oil. As shown in Figure 5f, the ^1^H NMR characteristic peaks of bio-oil are the carboxylic acid (-COOH) proton at 10.4 ppm, possibly due to an exchange reaction, which is not a clear broad peak, we draw a circular arrow to locally enlarge at 10.4ppm, and the characteristic peaks of methylene (-CH_2_-) proton and methyl (-CH_3_) that appear at 2.7 ppm and 2.05 ppm. The peak position of the ^1^H NMR is consistent with that of other research [62,63]. As shown in Figure 5g, the ^13^C NMR characteristic peaks of bio-oil are carbonyl carbon (C=O) at 178.9 ppm (carboxylic carbonyl), methylene carbon (-CH_2_-) at 38.2 ppm, and methyl carbon (-CH_3_) at 30.2 and 28.6 ppm. The peak position of the ^13^C NMR is also consistent with that of other research [62,63]. By comparing the ^13^C NMR spectra of bio-oil in Figure 5g with standard LA in Figure 5h, four characteristic peaks appear at the same position, which means that LA was detected in bio-oil.

The calorific value of liquid products directly determines their industrial value in the fuel system. To evaluate the catalytic performance of the photocatalyst in converting cellulose into high calorific value liquid products, the elements and calorific values of the liquid products were analyzed, and the results are shown in Table 3.

Compared to the H content (5.87%) and O content (57.53%) of the original cellulose, the liquid product obtained by photothermal catalysis has a higher proportion of H (8.64%) and lower O content (44.39%). The H/C ratio increases and the O/C ratio decreases as cellulose is depolymerized by photothermal catalysis, and the calorific value reaches 21.01 MJ/kg, which is significantly higher than that of the original cellulose (13.17 MJ/kg). Compared to the calorific value of bio-oil (15.50 MJ/kg) obtained by liquefying cellulose under subcritical conditions (260 °C), the calorific value of the bio-oil obtained by this method increased by 42% (6.51 MJ/kg). In addition, it is very close to the calorific value of bio-oil produced by pyrolysis of corn straw at 500 °C (22.38 MJ/kg) [65], but this study needs the low reaction temperature (220 °C), indicating that under the photothermal catalytic system, Pt/TiO_2_-CNT can promote the cleavage of chemical bonds in cellulose. Cellulose was successfully converted into bio-oil under mild conditions through dehydration, hydrolysis, and other reactions.

### 3.4. Photothermal Catalytic Condition

**Figure 6:**
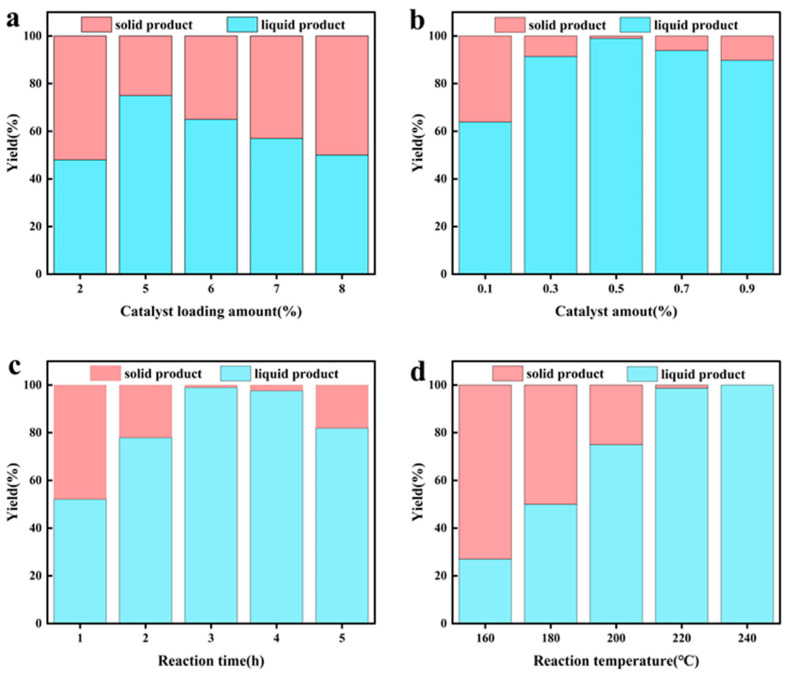
Details of the single factor test by adopting Pt/TiO_2_-CNT as the catalyst: (**a**) catalyst loading amount; (**b**) catalyst amount; (**c**) reaction time; (**d**) reaction temperature.

The effects of Pt loading, catalyst amount, reaction time, and reaction temperature on the degradation performance of cellulose were investigated by using Pt/TiO_2_-CNT as the catalyst, and the results are shown in Figure 6. As shown in Figure 6a, when the loading of Pt in the catalyst increased from 2 wt% to 5 wt%, the yield of the liquid fuel gradually increased from 47.8% to 75.2%. However, as the metal loading in the catalyst increased to 8 wt%, the yield of liquid fuel decreased to 50%, which may be due to excessive metal loading, which leads to the formation of metal ions and cellulose to form complexes, gradually forming agglomerates and generating more solid products [3]. As shown in Figure 6b, as the addition ratio of catalyst and cellulose increased from 0.1 to 0.5, the cellulose conversion rate increased from 63.9% to 99%, but the degradation rate of cellulose decreased as the catalyst amount further increased. Because increasing the amount of catalyst can provide more reaction sites and accelerate the degradation rate of cellulose, however, more byproducts may be produced if the excess catalyst is added [22]. As shown in Figure 6c, the cellulose conversion rate first increases and then decreases with the increase in reaction time, reaching a maximum value of 98.7% at 3 h. However, as the reaction time further prolongs, the cellulose conversion rate gradually decreases. Because the reaction progresses more fully and the yield of monomer products increases as time increases, excessive reaction time can lead to an accelerated rate of byproduct generation. As the reaction temperature increased from 160 °C to 240 °C, the cellulose conversion rate increased gradually. The conversion rate of cellulose reached 99.44% at 220 °C, which can be considered complete degradation. Further increasing the temperature will lead to an increase in energy costs. These phenomena indicate that the reaction temperature can effectively promote the depolymerization process of cellulose.

### 3.5. Proposed Reaction Mechanism

**Figure 7:**
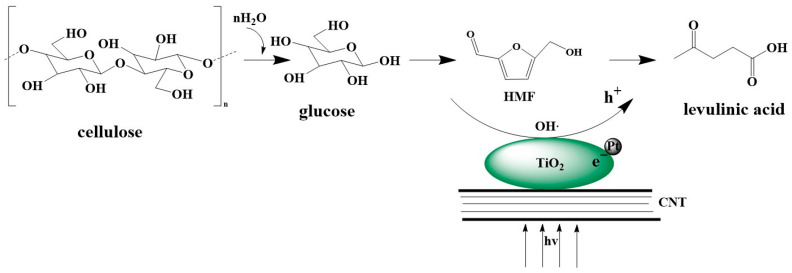
Rreaction pathway diagram of photothermal catalytic reaction mechanism of cellulose on Pt/TiO_2_-CNT catalyst.

Photothermal catalytic degradation of cellulose mainly relies on hydroxyl radicals to promote the depolymerization of cellulose. The reaction mechanism is speculated as shown in Figure 7. Firstly, titanium dioxide, by absorbing sunlight, can make water molecules produce hydroxyl radicals (HO) on the surface. The hydroxyl radicals take away the hydrogen atoms of C_4_ on the glucopyranose ring to produce a carbon radical and then combine with a molecule of oxygen to obtain the superoxide radicals. Meanwhile, this is accompanied by the cleavage of-1,4 glycosidic bonds, and a C_1_ radical and an alkoxy radical are obtained, which will combine with hydroxyl radicals to generate hydroxyl and alkyl superoxide, respectively. The alkyl superoxide can react with a molecule of water to obtain hydroxyl, glucose, and partially degraded cellulose fragments. Glucose is further degraded under acidic conditions, and glucose molecules combine with H^+^ to form glucose cations, dehydrate and remove H to generate furans, and then remove two molecules of water to generate 5-hydroxymethylfurfural (5-HMF). Since the oxidation reaction of hydroxyl radicals produced by TiO_2_ photocatalysis lacks selectivity, the generated 5-hydroxymethylfurfural will further undergo a degradation reaction, which can maximize the degradation of HMF by promoting a nucleophilic attack at its carbonyl group. Hydroxyl ions can effectively attack carbon cations in HMF and then break the C-O bond [60]. The hydroxyl radicals capture the electrons on the double bonds of the furan ring, rearrange to form a carbon radical, and then combine with the hydroxyl radicals to dehydrate and form a double bond. The terminal hydroxyl group combines with H^+^ in the acid solution to lose one molecule of water to obtain an unstable intermediate product and then rearranges to obtain LA [66].

## 4. Conclusions

To improve the harsh operating conditions of high temperature and high pressure required for converting cellulose into bio-oil, reduce the reaction cost, and improve the conversion rate and product selectivity of cellulose degradation, this study studied the photothermal catalytic technology of degrading cellulose to prepare the bio-oil. The effects of catalysts prepared by TiO_2_-CNT loaded different metals on the photothermal depolymerization of cellulose were discussed, and the photothermal catalytic process condition of cellulose was studied with Pt/TiO_2_-CNT as the catalyst. The reaction mechanism of photothermal catalytic degradation of cellulose was deduced. The main results of the study are as follows.

(1) A synergistic effect between heating and photocatalysis is present in cellulose degradation. TiO_2_-CNT loaded Pt exhibits the best cellulose photocatalytic performance, followed by Pd, Ru, Au, V, and Cu.
(2) Photothermal catalysis increased the H/C ratio and decreased the O/C ratio of the liquid product. The calorific value of the liquid product was 21.01 MJ/kg, which was significantly higher than that of the original cellulose (13.17 MJ/kg) and that in other research (15.50 MJ/kg). Cellulose is efficiently converted into bio-oil under mild conditions.
(3) Using Pt/TiO_2_-CNT as a catalyst, the conversion of cellulose bio-oil reached 99.44%, and the selectivity of LA reached 44.4% at 220 °C for 3 h.

This study significantly improved the bio-oil conversion rate and selectivity of cellulose with a low reaction temperature and short time. The economic feasibility of the catalyst system should be analyzed in subsequent research. This will help to reduce the production cost of cellulose conversion, improve the selectivity of products, and promote the further industrial application of lignocellulose to prepare fuel oil, thereby reducing the environmental pollution caused by the large consumption of fossil fuels.

## Figures and Tables

**Table 1:** Comparison of cellulose degradation performance under different technologies.

Samples No.	Catalysts ^1^	Illuminating ^2^	Heating	Conversion (%)
1	No catalysts	NO	NO	0
2	No catalysts	YES	NO	0
3	No catalysts	YES	YES	70.3%
4	TiO_2_-CNT	NO	YES	76.2%
5	TiO_2_-CNT	YES	NO	13.6%
6	TiO_2_-CNT	YES	YES	78.1%
7	Pt/TiO_2_-CNT	YES	NO	22.5%
8	Pt/TiO_2_-CNT	NO	YES	87%
9	Pt/TiO_2_-CNT	YES	YES	98%

^1^ The ratio of cellulose to catalyst was 2:1. ^2^ The temperature can be increased by 1 to 5 °C under light.

**Table 2:** BET test results of the catalysts with different metal loads.

Catalysts	Surface Area ^1^, m^2^/g	Pore Volume ^2^, cm³/g	Average Pore Size ^3^, nm
TiO_2_-CNT	60.98	0.37	22.13
Ag/TiO_2_-CNT	102.56	0.54	19.06
V/TiO_2_-CNT	212.28	0.72	12.76
Pt/TiO_2_-CNT	108.92	0.63	21.49
Pd/TiO_2_-CNT	99.41	0.60	22.24
Au/TiO_2_-CNT	112.10	0.60	18.83
Cu/TiO_2_-CNT	87.95	0.56	22.85
Ru/TiO_2_-CNT	108.57	0.58	18.88

^1^ Multi-point BET method was used for calculation. ^2^ Calculated by BJH adsorption cumulative pore volume. ^3^ According to BJH adsorption average pore size calculation.

**Table 3:** Elemental content and calorific value analysis of cellulose and liquid products.

Sample	Elemental Content (wt.%)						HHV (MJ/kg)
	C	H	O	N	H/C	O/C	
Cellulose	40.87	5.87	57.53	0.003	0.14	1.40	13.17
liquid	49.85	4.77	45.33	0.27	1.14	0.69	15.50 [64]
liquid	46.65	8.64	44.39	0.33	0.19	0.95	21.01

## Data Availability

Data is contained within the article.
